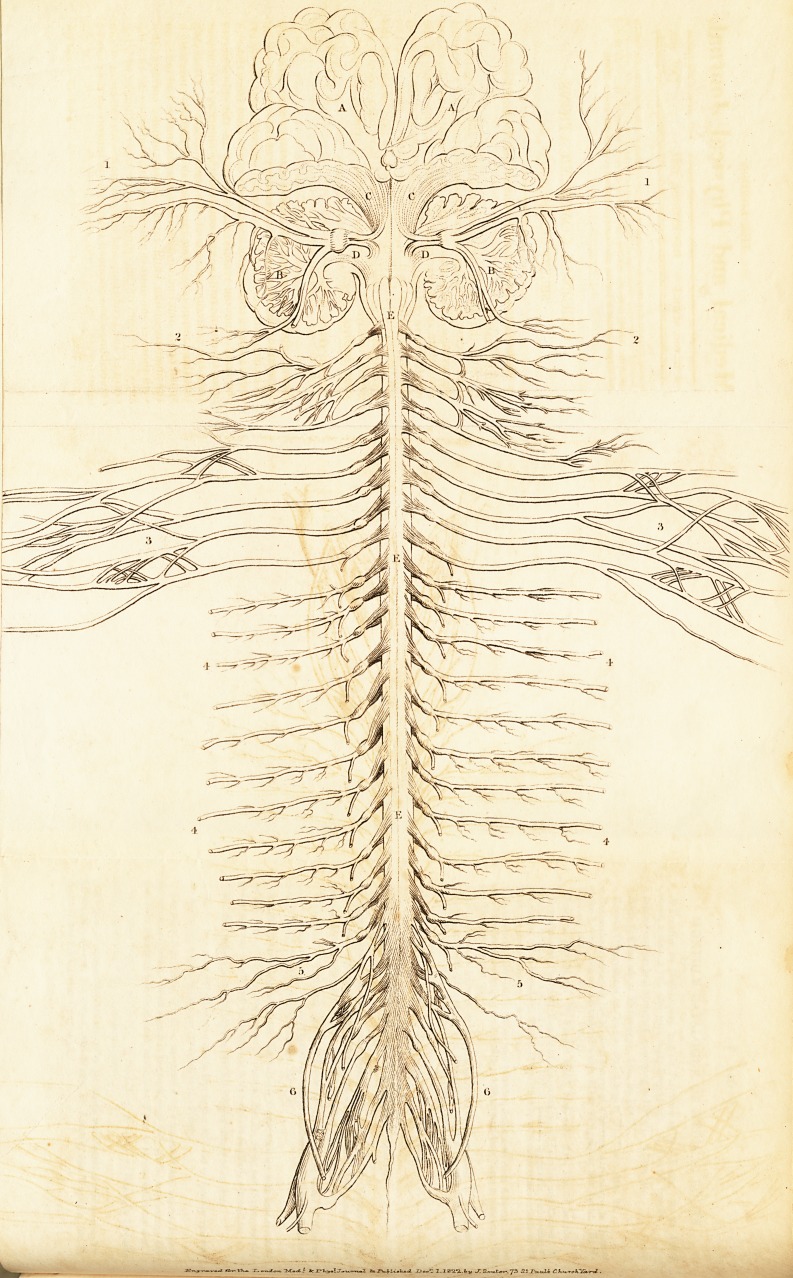# Mr. Shaw on the Nervous System

**Published:** 1822-12

**Authors:** J. Shaw

**Affiliations:** Albany


THE LONDON

### Medical and Physical Journal.

6 OF VOL. XLVIII.] DECEMBER, 1822. [No. 286.

For many fortunate discoveries in medicine, and for the detection of numerous errors, the world is indebted to the rapid circulation of Monthly Journals; and there never existed any work, to which the Faculty, in Europe and America, were under deeper obligations, than to the Medical and Physical Journal of London, now forming a long, but an invaluable, series.—RUSH.

### ORIGINAL COMMUNICATIONS, SELECT OBSERVATIONS, &c.

*To the Editors of the London Medical and Physical Journal.*

GENTLEMEN,

I TRUST the date of this communication will be regarded as some excuse for the imperfect manner in which I have executed the promise I lately made to you, as you must know the extensive nature of my engagements during the first month of the winter course of Lectures. I hope, however, that the following sketch, imperfect as it is, may enable such of your readers as are interested in the discussion, to form a general idea of the investigations in which Mr. Bell and I have been recently engaged, relative to the nervous system. I remain, Gentlemen, your obedient servant,

J. SHAW.

*Albany; October* 28, 1822.

ABOUT fifteen years ago, Mr. Bell's attention was particularly directed to the subject of the physiology of the nervous system. The circumstance which first attracted his notice was the difference in the distribution of the nerves of the head from those of the body, and the remarkable fact that all the spinal nerves arose by double roots,—viz. one from the anterior, and another from the posterior column of the spinal marrow. Observing that this form of origin was the same in all animals possessing a spinal cord, and finding that the observations he had made on the anatomy of the brain in the lower animals, corresponded with those of the most distinguished anatomists,—viz. that the anterior column of the spinal marrow was continuous with the crura of the cerebrum, and the posterior with the crura of the cerebellum,—he conceived that, by experiments on the roots of these nerves, he might discover the functions of the two columns, and perhaps, through them, arrive at a more accurate knowledge of the relations and individual uses of the cerebrum and cerebellum. The experiments were made,* and were followed by the same results as those which were lately performed by M. Magendie, and of which notice is taken in your Journal for October. An account of these experiments was printed and distributed among Mr. Bell's scientific friends in 1809. The same essay contains several speculations, which later investigations have proved to be in a great measure correct. I shall here quote a few passages from the essay, to prove that many of the views he has lately published, through the medium of the Philosophical Transactions, have for a long time occupied his attention.

"I took this view of the subject:—The medulla spinalis has a central division, and also a distinction into anterior and posterior fasciculi, corresponding with the anterior and posterior portions of the brain. Further, we can trace down the crura of the cerebrum into the anterior fasciculus of the spinal marrow, and the crura of the cerebellum into the posterior fasciculus. I thought that here I might have an opportunity of touching the cerebellum, as it were, through the posterior portion of the spinal marrow, and the cerebrum through the anterior portion. To this end I made experiments, which, though they were not conclusive, encouraged me in the view I had taken.

"I found that injury done to the anterior portion of the spinal marrow convulsed the animal more certainly than injury done to the posterior portion; but I found it difficult to make the experiment without injuring both portions.

"Next considering that the spinal nerves have a double root, and being of opinion that the properties of the nerves are derived from their connexions with the parts of the brain, I thought that I had an opportunity of putting my opinion to the test of experiment, and of proving, at the same time, that nerves of different endowments were in the same cord, and held together by the same sheath.

"On laying bare the roots of the spinal nerves, I found that I could cut across the posterior fasciculus of nerves, which took its origin from the posterior portion of the spinal marrow, without convulsing the muscles of the back; but that, on touching the anterior fasciculus with the point of the knife, the muscles of the back were immediately convulsed.

"Such were my reasons for concluding that the cerebrum

* I may observe that, previous to having made these experiments, Mr. Bell entertained the opinion that the anterior column of the spinal marrow was different in function from the posterior, and that, through it, the simple voluntary power of moving particular parts was conveyed. He deduced this from observing that the two nerves, which are generally supposed to be purely motors,—viz. the third, or motor oculi, and the ninth, or motor linguæ, arose from the anterior fasciculus.

and the cerebellum were parts distinct in function, and that every nerve possessing a double function, obtained that, by having a double root. I now saw the meaning of the double connexion of the nerves with the spinal marrow; and also the cause of that seeming intricacy in the connexion of nerves throughout their course, which were not double at their origins."

Although the experiments above alluded to, were sufficient to show that a part which is endowed with the power of performing more than one function must have distinct nerves for each function, still they were so difficult to perform, and so liable to error, that Mr. Bell would not found any theory upon them. He therefore, for a considerable time, pursued the inquiry in a different manner.

During many succeeding seasons, he was engaged with his house-pupils in the investigation of the comparative anatomy of the brain, and in making dissections and preparations to illustrate the distinction between the different nerves of the body. The result of the examination of the minute structure of the nerves, proved that those which arose from the spinal marrow by double roots were very different in texture from certain other nerves, (the par vagum, for example;) and, upon this discovery, Mr. Bell considered himself entitled to state, in his public lectures, that there must be two distinct classes of nerves independent of the sympathetic.

Having once formed this idea upon such satisfactory evidence, he was unremitting in his researches, in all of which he was assisted by his pupils. The next circumstance, which more particularly engaged his attention, was the fact of the nerves which supplied the limbs being very simple in their arrangement, compared with those which are distributed to the head, neck, chest, and abdomen. This consideration led him to the idea that he might be able to unravel the seeming intricacy of this part of the nervous system, by means of comparative anatomy: accordingly, he directed his attention more particularly to the distribution of the nerves in the different classes of animals; and, by this manner of investigating the subject, he was at length enabled to come to the conclusion which is offered in his first paper to the Royal Society.

"When we minutely and carefully examine the nerves of the human body, and compare them with those of other animals, a very singular coincidence is observed between the number of organs, the compound nature of their functions, and the number of nerves which are transmitted to them. No organ, which possesses only one property or endowment, has more than one nerve, however exquisite the sense or action may be; but if two nerves, coming from different sources, are directed to one part, this is the sign of a double function performed by it. If a part or organ have many distinct nerves, we may be certain that, instead of having a mere accumulation of nervous power, it possesses distinct powers, or enters into different combinations, in proportion to the number of its nerves."

To show that, previous to having taken advantage of the proofs afforded by comparative anatomy, Mr. Bell had founded a theory somewhat similar, on the observation of the anatomy of the nerves in the human body, I shall quote some passages from the essay already alluded to. It is to be understood, however, that they are not brought forward as correct views, but merely to show how long the subject has been matter of consideration.

"The spinal nerves being double, and having their roots in the spinal marrow, of which a portion comes from the cerebrum and a portion from the cerebellum, they convey the attributes of both grand divisions of the brain to every part; and therefore the distribution of such nerves is simple, one nerve supplying its destined part. But the nerves which come directly from the brain, come from parts of the brain which vary in operation; and, in order to bestow different qualities on the parts to which the nerves are distributed, two or more nerves must be united in their course, or at their final destination."

"Hence it is that the first nerve must have branches of the fifth united with it; hence the portio dura of the seventh pervades every where the bones of the cranium, to unite with the extended branches of the fifth: hence the union of the third and fifth in the orbit: hence the ninth and fifth are both sent to the tongue: hence it is, in short, that no part is sufficiently supplied by one single nerve, unless that nerve be a nerve of the spinal marrow, and have a double root,—a connexion (however remotely) with both the cerebrum and cerebellum.*

"Such nerves as are single in their origin from the spinal marrow, will be found either to unite in their course with some other nerves, or to be such as are acknowledged to be peculiar in their operation.

"Understanding the origin of the nerves in the brain to be the source of their powers, we look upon the connexions formed betwixt distant nerves, and upon the combination of nerves in their passage, with some interest; for, without this, the whole is an unmeaning tissue. Seeing the seeming irregularity in one

* By this quotation, it will be seen that, at the time it was written, Mr. Bell had not discovered the identity of the fifth and the spinal nerves, a discovery which has done more than any other to unravel the intricacy of the nerves of the head: still the principle holds good; for, although the fifth pair bestows sensibility and motion, yet other nerves are necessary when the motions of the features, the nose, lips, eye-lids, and even the ball of the eye, become subject to a different influence from that which governs the common motions and common sensibilities of the frame.

subject, we say it is accident; but, finding that the connexions never vary, we say only that it is strange, until we come to understand the necessity of nerves being combined, in order to bestow distinct qualities on the parts to which they are sent."

It was not, however, until about three years ago that Mr. Bell found himself entitled to offer the conjecture, that several nerves, which had hitherto been supposed to be of the same character, were not only different in structure but also in function; and this he deduced, not from experiments, but from rigid inquiries into the comparative anatomy of the nervous system. The same mode of inquiry also induced him to suspect that the intricacy of the nerves of the head and trunk, when compared with those of the limbs, depended in a great measure on the particular form of the respiratory organs, and on their combinations with the different functions of the throat, heart, and stomach.

It became now highly interesting to discover how far these views deduced from anatomy, would be substantiated by experiments. We had already seen that the results of cutting the fibrils of the spinal nerves which arise from the posterior column of the spinal marrow, were very different from those presented when the fibrils connected with the anterior column were divided. We then looked for two nerves which lay nearly in the same situation and supplied the same parts, but the origins of which were from different portions of the brain. We fixed upon the 5th and portio dura. The consequences of cutting the one nerve were so different from those following the division of the other, that there now remained no question of the general correctness of the theory, that there are two distinct classes of nerves besides the sympathetic. But this experiment was the means of leading to a very curious discovery, and to a knowledge of facts which every one will allow to be most important to the sciences of medicine and surgery. Part of these are already before the public, and particularly the observations on the union of the functions of many parts of the face with the respiratory organs of the chest and throat, through the medium of the portio dura. The facts which could be adduced in support of this particular part of his theory were so many, and so striking, that Mr. Bell, well aware of the reluctance of the scientific world to listen to any new theory of the nervous system, unless some obvious and conclusive facts were offered in support of it, selected, as his first paper to the Royal Society, the observations which he had made on the Nerves of the Face; for, by the facts which he could bring forward, he hoped to establish the general correctness of the most important part of his discoveries, that "no organ which possesses only one property, or endowment, has more than one nerve, however exquisite the sense or action may be; and if two nerves, coming from different sources, are directed to one part, this is the sign of a double function performed by it."

The few criticisms which have been made on this paper are such as would naturally proceed from those who were not aware that the principal object of it was to direct the attention of the scientific world to a great question; and, as the writers of those criticisms have been led into errors regarding the whole theory, in consequence of their having taken only very partial views of the question, I shall here offer a few observations on the manner in which the conclusions, contained in the papers already published, were deduced.

The facts of comparative anatomy distinctly led to the conclusion, that, if the mouth was not furnished with moveable lips, or cheeks, no branches of the portio dura passed to it, although it might receive many branches from the fifth; and, indeed, the anatomy of the nerve in many fishes furnishes examples of the truth of this. The next fact observed was, that, where the eyelids are immoveable, no branches of the portio dura are sent to them, though part of the orbit is furnished with branches of the fifth pair. These facts alone were sufficient to entitle us to consider the portio dura as a superadded nerve, and that its presence depended on the existence of organs which might be considered as superadded or additional. The nerve was therefore (previous to the performance of any experiments,) arranged as one of the superadded class: that is, as one of the class distinct from the spinal nerves; and it was stated that its existence in an animal depended on the same rules as that of certain other nerves,—as, for instance, the phrenic, which, it is well known, does not exist unless there be a diaphragm. By pursuing the comparative anatomy of the portio dura farther, we discovered that there was a very striking resemblance between it and those branches which supply certain parts of the organs of respiration. Thus, in fishes, we found a nerve, similar in origin to the portio dura, pass to the muscles moving the opercula of the gills; and in some birds, as in the duck, we found the portio dura so intimately connected with the eighth, and having so little connexion with the auditory nerve, that, previous to the performance of any experiments, we concluded that the portio dura was not only of the superadded class, but that it must also be connected with the function of respiration. This latter view was proved to be correct by the first experiment made; for, on cutting the nerve, the power of inflating the nostrils during respiration was destroyed. Thus two circumstances were made out by a regular train of reasoning, founded on the facts furnished by comparative anatomy. There was, however, still more to be discovered in the same manner: in the first experiment made, it was observed that the eye-lids were also paralyzed by cutting the nerve. The fact already noticed, that, where there were no moveable eye-lids, there was no distribution of the portio dura on the orbit, prevented us from being surprised at this result: but, to explain the connexion between the organs of respiration and the eye-lids, required a little consideration. The observations which are already before the public, proving that the expression of the face, nay, of the whole body, has such an intimate connexion with the organs of respiration, afford sufficient explanation why the same nerve should combine the motions of the eye-lids and forehead with the acts of respiration, and with the motions of expression performed through the muscles of the nose and mouth.*

The next fact which naturally excited our attention was, that there were branches of the portio dura distributed not only on the external ear, but also to the muscles within the cavity of the tympanum. It was also observed, that, if the trunk of the portio dura was cut near its exit from the styloid hole, that the muscles of the external ear were paralyzed. If this be taken into account with the observation, which must be familiar to every one, of the similarity of the expression in the *cocking* of the ear in the dog to the state of the eye-lids and cheeks when the animal is excited, it should be sufficient proof of the portio dura being also for the supply of the superadded parts of the ear, which may be considered as analogous to those of the eye. Though it may be impossible, or at least very difficult, to make an experiment by which we should see the effect produced on the internal muscles of the ear, still, after such a series of observations, it is not too much to suppose, that the branches which pass to the muscles within the tympanum are for combining their actions with those of the external muscles of expression; for I think it will be allowed by every one, that there is not only a sense of alacrity in the ear during the excited state of the muscles of expression, but that we have also a certain power over these muscles, corresponding to the command we have over the

* "It will be asked, why a nerve called respiratory should go to the ear and eye? First let us inquire, does it belong to the frame of animal bodies that there shall be in them indications of passion? If it be admitted that this is the case, we here learn, in addition, as the portio dura is the nerve of respiration, so is it the grand nerve of expression, not only in man, but in brutes also: all that excitement seen in a dog's head, his eyes, his ears, disappears if this nerve be cut. The respiratory nerve being cut across in a terrier, the side of the face was deprived of all expression, whether he was made to crouch, or to face an opponent and snarl. When another dog was brought near, and he began to snarl and expose his teeth, the face, which was balanced before, became twisted to one side, —to that side where the nerve was entire; and the eye-lids being, in this state of excitement, very differently affected, presented a sinister and ludicrous expression."—*Philosophical Transactions,* July 1821.

muscles of the face. This we are conscious of exerting in a certain degree, when we endeavour to counteract the effects of the concussion of the air produced by the discharge of a large cannon. In support of this, (which however, I allow, is a mere hypothesis,) I shall offer a fact observed in comparative anatomy. In the cod-fish, where there are neither moveable lips nor moveable eye-lids, nor external ear, nor ossiculi corresponding to those of the tympanum, the portio dura does not take the same course that it does in animals furnished with those parts, but goes directly to the muscles of the gills. Indeed, even in some birds we have similar evidences. Let us take the anatomy of the duck: as in this bird there is little or no external ear, and little or no motion in the tympanum, we may perhaps be permitted to conclude, that it is in consequence of this, that the portio dura does not go along with the auditory nerve into the ear, but makes its exit by a distinct foramen, to supply the only parts about the head which the duck has in common with the quadrupeds. It was after ascertaining these facts by anatomy, and after forming juster opinions of the uses of the portio dura, that it became important to examine whether by experiment we should find that there was as much difference in the functions of the portio dura and of the fifth, as there was proved by anatomy to be, not only in their origins, but also in their structure, appearance, and distribution.

The first experiment made, was to cut the portio dura on one side: the nostrils, lips, and eye-lids, were all immediately paralyzed; but the sensibility of the paralytic part was not in the slightest degree diminished, and, when food was presented to the animal, it ate easily, being enabled to pick up the oats with the lips, and to move its jaws as well as before the nerve was cut.

On cutting the infra-orbital nerve of the other side, no effect whatever was produced on the motions of the nostrils, but the sensibility of the parts was destroyed. Here then was sufficient proof of the two nerves being as different in function as they are in origin and structure. A further proof of their difference was observed, in the animal appearing to suffer much less pain when the portio dura was cut, than when the fifth was divided. A circumstance was also noted immediately after the animal was killed, and while there yet remained a power of action in the muscles, which, perhaps, more than any other fact, establishes the difference between the functions of the branches of the two nerves on the face, and at the same time involves one of the most curious questions in physiology. Although the portio dura had been divided for two days, and thus, according to the received opinions, separated for that time from the influence of the brain, still, when the end connected with the muscles was stimulated, convulsions took place; while no similar effect was produced by stimulating the fifth. I state this here, merelyto show what an extensive field for observation on the functions of the nerves is now opened; for we know that if the fifth nerve had been divided nearer the brain, somewhat similar results would have followed upon its being stimulated; but, as farther observations would lead me to the question of the origins of the fifth nerve, I shall at present only remark, that the results of some operations, in which the portio dura in the human body has been cut, afford very curious proofs of the compound nature of the functions of those branches of the fifth pair which supply the lips. Perhaps, indeed, to prove the lip to be an organ of such a compound nature, that, although we say it receives only two nerves, the portio dura and the fifth, there may be in these two nerves several fibrils possessing distinct powers.

When the animal was let loose after the infra-orbital nerve was cut, it was observed that it could no longer eat, although it had fed very well when only the portio dura of the opposite side was cut.

This result naturally interested us very much, but, by a singular combination of circumstances, the cause of this was not then discovered: when, however, all the bearings of the experiment were understood, it was shown to be much more important and curious than we at first supposed; and that it established more strongly certain conclusions originally drawn from anatomical observations, and which I shall presently adduce.

As we found that the animal was not deprived of the power of feeding by the division of the portio dura, we endeavoured, by the next experiment, to discover how far the power of raising the corn with the lips would be affected by cutting the branches of the fifth. The infra-orbital nerves of both sides were therefore cut in another ass: upon the animal being let loose, he could no longer pick up the oats with the upper lip, but gobbled them up by a combined effort of the jaws, lower lip, and tongue; but the actions of the nostrils during respiration continued entire, although their sensibility was destroyed. Here then, were two experiments followed by very distinct results.

In the first experiment, it was found that, when the portio dura was cut, the actions of the nostril during excited respiration were destroyed; and, when the infra-orbital nerve of the other side was cut, that the animal could no longer eat. In the second experiment, although the sensibility of the nostrils were destroyed, their actions during respiration continued entire. It was, moreover, observed that the animal could no longer pick up the oats with its lips. Hence, by this experiment, it was distinctly proved that, on cutting these two branches of the fifth, the powers of feeding were impaired; and upon this we naturally concluded, that if all the branches of the fifth nerve were cut, that the animal would be deprived of the power of feeding. Experiments, made since, have fully established the correctness of this deduction; but it was not till lately, that we were able to show the very curious cause of the loss of power in feeding, consequent upon cutting the infra-orbital branches of the fifth.

The effects produced upon the nostril, as an organ of respiration, and upon the muscles of the face, as organs of expression, were so evident on cutting the portio dura of one side, that we thought it a needless cruelty to cut that of the other; and it was in consequence of this, that we were for a long time not aware of the very important assistance which such an experiment would have afforded, not only in explanation of the preceding experiments, but also in proof of a fact which we had been previously led to suspect from the study of comparative anatomy,—viz. that the portio dura was a nerve as similar in function as it is in origin and form to the branches of the eighth pair.*

Particular reasons at length induced me to perform this experiment, and then several circumstances, which had been previously a little obscure, were explained; for, immediately on cutting the nerve on both sides of the face, the lips became so paralyzed, (although they still retained their sensibility,) that the animal could no longer use them in raising its food. Thus we now had experiments to show, that if both the infra-orbital nerves of the fifth were cut, or if the portio dura of each side was cut, that the power over the lips in feeding was destroyed.

These facts are important, as they make it easy to explain the results of the first experiment, (by which the animal was also deprived of the power of picking up the oats with its upper lip;) for we can understand that, by cutting the portio dura of one side, the corresponding side of the lip would be deprived of its power of motion; while, by cutting the infra-orbital nerve of the other side, we should deprive it of its sensibility; and thus, the lip being deprived of motion on one side, and sensibi-

* When the portio dura of one side is cut in experiments or in operations, or when it is paralyzed, the effect upon the muscles of the nostril, as organs of respiration, and upon the muscles of the face, as organs of expression, is distinctly marked; while the effect upon the same muscles, as far as they assist in mastication, by pushing the morsel from one side of the mouth to the other, is so slight, that the influence of the portio dura over the muscles in performing this function was not, even after very careful observations, fully discovered until both portio duras were cut in the same animal. The effect produced upon the muscles immediately in the attempt to feed was so evident, that there could no longer exist any question of this nerve having an influence over the muscles of the face, similar to that of the branches of the eighth pair over the pharynx and larynx.

lity on the other, would become as useless as if both infra-orbital nerves, or both portio duras, had been divided. When we move a morsel in the mouth during mastication, we are apt to say that, to do so, it is only necessary that the muscles should be endowed with a power of motion. But this is not perfectly correct. There are two powers in operation; and the function would be destroyed, either by depriving the tongue and cheeks of sensibility, or their muscles of the power of motion: for, if the mouth was deprived of sensibility, the morsel not being felt, no direction could be given to the muscles, and consequently it could not be moved, and successively put under the operation of the teeth and jaws. This is what takes place when the lips are deprived of their sensibility; for although, by smelling, a creature is directed to the food, yet, when the lips touch it, there being no sensation, there is no direction given to the muscles of the lips to gather it; and hence, after ineffectual endeavours, the animal touches the morsel with the tongue, and licks it up like a dog.

The experiment of cutting the portio dura on both sides also enabled us to establish another important fact,—viz. the similarity of the functions of the portio dura to those of the branches of the eighth pair, which we had been already led to suspect, on observing the intimate connexion which there is in some animals between the origins of the two nerves. By cutting the portio dura of each side, not only were the respiratory functions of the muscles of the face destroyed, but also their power of assisting in mastication, being an effect analogous to the consequence of cutting the branches of the eighth pair; for, by that division, there not only follows the destruction of the larynx, as an organ of respiration, but also the deterioration of the actions of the pharynx, as part of the apparatus necessary to deglution.

Instead of now going into a separate detail of the facts of anatomy, and of the experiments by which certain other nerves were shown to be as distinct in character and function from those which arise from the spinal marrow, as the portio dura is from the fifth, I shall exhibit the characters of the two classes, with the assistance of the plates which have been already published in the "Manual of Anatomy." In Plate I. we have a plan of the cerebrum and cerebellum, and of their crura, which correspond to the columns of the spinal marrow;—the crura of the cerebrum to the anterior, and the crura of the cerebellum to the posterior column. In the same plate we have also a plan of the origins of all the spinal nerves, and of the fifth, or trigeminus.

The characters of the spinal nerves may be thus defined:—they have all double origins,—they have all ganglia on one of their roots,—they go out laterally to certain divisions of the body,—they are all compound nerves, being at the same time muscular nerves, ordering the voluntary motions of the frame, and bestowing sensibility on the surfaces of the body,—they are exquisitely sensible, and they pervade every part. These are also the nerves which are affected in the common cases of hemiplegia; and when, in experiments on animals, or in operations on the human body, the trunk of one of these nerves is divided, the muscles to which it goes are deprived of the power of executing certain motions; and the sensibility of the part of the skin to which the nerve is distributed is also destroyed. But if only one of the origins be divided, (the anterior,) the power over the motions of the muscle will alone be destroyed, while the sensibility of the part will continue perfect; but this will also be destroyed if the other origin (the posterior,) be divided. These properties of the spinal nerves, with the exception of the difference in the effect produced by cutting the two origins, have been allowed by anatomists of all ages. It remains, however, that I should now prove the correctness of a discovery, which is most important to the anatomist,—viz. the similarity, in every respect, of the nerve commonly called the fifth of the brain, to the spinal nerves. To do this, I shall nearly repeat part of the observations which I made in my last communication to this Journal.

1. That the head and face, having many parts in every respect similar to the neck, trunk, and limbs, must have corresponding nerves.

2. That the manner in which the spinal nerves and the fifth arise by double origins, (as may be seen in the plate,) is very similar.

3. That the two origins of the fifth are united by a ganglion exactly of the same shape and character as those which unite the two origins of the spinal nerves.

4. That the manner in which the branches of the fifth are distributed, and those of the spinal nerves, is the same.

And, lastly, that the same kind of connexion exists between the fifth and the sympathetic, as between the latter and the spinal nerves.

In their morbid affections, the similarity also holds good:—thus, in the common cases of hemiplegia, the spinal nerves and the branches of the fifth are similarly affected. In this disease, the voluntary power over the limbs, and the sensibility of the side affected, are generally destroyed; but in some cases the voluntary power is lost, and the sensibility continues unimpaired, or *vice versa*. This variety also occurs on the face; for the jaw will drop, and there will be all the marks of paralysis, while the sensibility of the skin, and the sense of taste, will continue entire.

In experiments on the nerves of the spine and on the fifth, we meet with the same results. If, as in the operation which is now frequently performed on the nerves of the horse's foot, we cut a spinal nerve after the branches are given off to the muscles moving the part, we shall destroy only the sensibility of that part; but, if we cut the nerve nearer to the brain, we shall not only destroy the sensibility, but also the power of motion. The same happens in experiments on the fifth; for, if we cut a branch which is principally distributed to the skin of the lips, we shall destroy the sensibility of the part, but impair the power of mastication only in a slight degree: but, if we divide the nerve further back, then we shall not only destroy the sensibility of the skin, as in the first experiment, but also cut off the power by which the jaws are moved. I cut a branch of the fifth upon the face: the sensibility of the corresponding side of the lip was destroyed, but little paralysis ensued, excepting of certain actions of the orbicularis oris. I cut the nerve nearer the brain, and at a point previous to its having given off the branches to the other muscles: then the jaw fell, and the muscles of that side were powerless. I varied the experiment, by irritating the nerve where it lies in the spheno-palatine fissure, immediately after an animal was killed: the jaws then came together with much force, indeed, so as to nip my assistant's finger severely. This last experiment may be compared with the very common one of galvanizing the nerves which pass from the spinal marrow to supply the muscles of the extremities.

### EXPLANATION OF PLATE I.

A. A. Cerebrum.
B. B. Cerebellum.
C. C. Crura Cerebri.
D. D. Crura Cerebelli.
E. E. E. Spinal Marrow.

1. 1. Branches of the Fifth Pair, or Trigeminus, which are seen to arise from the union of the crura cerebri and crura cerebelli, and to have a ganglion at the roots.

2. 2. Branches of the Suboccipital Nerves, which have double origins and a ganglion.

3. 3. The branches of the four Inferior Cervical Nerves, and of the first Dorsal, forming the Axillary Plexus: the origins of these nerves are similar to those of the fifth and the suboccipital.

4. 4. 4. 4. Branches of the Dorsal Nerves, which also arise in the same manner.

5. 5. The Lumbar Nerves.

6. 6. The Sacral Nerves.

I shall now proceed to explain the second Plate, containing a plan of the nerves which have been considered superadded, or dependent upon the existence of parts that may be considered as additional to the original frame of the body. I shall afterwards offer (from high authorities in the profession,) proofs of the advantages already gained in the practice of medicine and surgery by the discovery that this class of superadded nerves is in every respect different from the original system of nerves, exhibited in Plate I.

**[The Editors are sorry to be obliged to defer the remainder of Mr. Shaw's communication until next month.]**

## Figures and Tables

**Figure f1:**